# Effects of Apolipoprotein E Polymorphism on Cerebral Oxygen Saturation After Traumatic Brain Injury

**DOI:** 10.3389/fneur.2020.539627

**Published:** 2020-11-12

**Authors:** Zhimin Wu, Senjie Xiong, Xiaochuan Sun, Quanhong Shi, Wei Dan, Yan Zhan, Yanfeng Xie, Li Jiang

**Affiliations:** ^1^Department of Neurosurgery, The First Affiliated Hospital of Chongqing Medical University, Chongqing, China; ^2^Department of Neurosurgery, University-Town Hospital of Chongqing Medical University, Chongqing, China

**Keywords:** TBI, APOE, regional cerebral oxygen saturation (rScO2), cerebral oxygen saturation, near-infrared spectroscopy (NIRS)

## Abstract

**Objective:** To investigate the effects of the apolipoprotein E gene (APOE) on the cerebral oxygen saturation of patients after traumatic brain injury (TBI).

**Methods:** Clinical data of 114 patients with TBI and 54 normal people were collected. The APOE genotypes of all subjects were determined by quantitative fluorescent polymerase chain reaction (QF-PCR). The regional cerebral oxygen saturation (rScO_2_) of TBI patients and normal people were monitored by near-infrared spectroscopy (NIRS).

**Results:** The mean rScO_2_ of patients was (55.06 ± 7.60)% in the early stage of TBI, which was significantly lower than that of normal people (67.21 ± 7.80)% (*P* < 0.05). Single-factor and multifactor logistic regression analyses showed APOEε4 was an independent risk factor that caused the early decline of rScO2 in TBI patients. Furthermore, in the TBI group, the rScO_2_ of APOEε4 carriers (52.23 ± 8.02)% was significantly lower than that of non-ε4 carriers (60.33 ± 7.12)% (*P* < 0.05). But in the normal group, no significant differences in rScO_2_ were found between APOEε4 carriers and non-carriers.

**Conclusion:** The rScO_2_ may be significantly decreased after TBI, and APOEε4 may be a risk factor for decreased rScO_2_ in the early stage of TBI.

## Introduction

Traumatic brain injury (TBI) is a common disease with high disability and mortality in the neurointensive care unit (NICU) (1–3). Cerebral blood flow often changes after TBI, which may lead to a series of pathological responses and irreversible brain damage (4, 5). As a result, TBI is often followed by ischemia and hypoxia, resulting in severe neurological impairment and death. Previous studies have shown that ischemia and hypoxia are strongly associated with poor outcomes (6, 7), and a decrease of cerebral oxygen saturation usually indicates the possibility of cerebral ischemia and hypoxia. Therefore, cerebral oxygen saturation monitoring is an important method to evaluate the condition of TBI patients during neurointensive care (8).

Near-infrared spectroscopy (NIRS) is a useful method to monitor regional cerebral oxygen saturation (rScO_2_) (9, 10). It has a good correlation with the jugular bulb oxygen saturation (SvjO_2_) which is considered a gold standard for brain oxygen metabolism (11) and is helpful for timely detection and correction of cerebral ischemia and hypoxia (12).

Apolipoprotein E gene (*APOE*) can affect the prognosis of TBI patients, and *APOE*ε*4*, a subtype of *APOE*, is considered as a risk factor for exacerbations of TBI outcome (13–16). However, the mechanism through which *APOE*ε*4* influences the prognosis of TBI patients remains unclear. In previous studies, we have found that *APOE* may affect the blood-brain barrier permeability of mice after TBI (17), but it is unknown whether it affects cerebral blood flow and cerebral oxygen saturation. Therefore, as soon as we discovered the phenomenon by accident in our daily clinical work that some TBI patients with *APOE*ε*4* had lower cerebral oxygen saturation, it aroused our interest immediately. So we proposed the hypothesis *APOE*ε*4* might be related to lower rScO_2_ as compared with *APOE*ε*2* and *APOE*ε*3* after TBI. To verify our hypothesis, we explored the relationship between *APOE* and rScO_2_ in the early stage of TBI in this study. The rScO_2_ was measured by NIRS, and the *APOE* types of the subjects were determined by quantitative fluorescent polymerase chain reac (QF-PCR).

## Materials and Methods

### Subjects

This is a retrospective study that complies with the ethical standards formulated by the Ethics Committee of the First Affiliated Hospital of Chongqing Medical University and has received its approval. The review batch number is 2019 Research Ethics (2019-026). Subjects included in this study were admitted to the hospital from March 2018 and to May 2019. For normal people and those patients who were conscious and cooperative, written informed consent were obtained from both patients and their legal guardians. For comatose patients, written informed consent was obtained from their legal guardians.

#### TBI Group

Inclusion criteria were patients with clear head injury history, admitted to hospital within 1–3 days after injury, and aged 15–65. Exclusion criteria were: (1) having a medical history that may affect cerebral oxygen saturation (e.g., TBI, cerebrovascular diseases, intracranial space-occupying diseases, encephalitis, psychosis or dementia, etc.); (2) with use of drugs that may affect cerebral oxygen saturation; (3) suffering from the respiratory circulatory system that seriously influences the cerebral oxygen saturation; (4) having serious scalp injury affecting the monitoring of cerebral oxygen saturation; (5) having serious multiple injuries complicated by severe dysfunction of other organs or systems (such as severe respiratory and circulatory dysfunction, electrolyte disorders, etc.).

According to the inclusion and exclusion criteria, TBI patients with medical histories that may affect cerebral oxygen saturation were excluded. All TBI patients were treated according to the guidelines of TBI (7, 18). In detail, for TBI patients, the possible factors that may influence the levels of rScO_2_ and blood oxygen saturation (SO_2_) were removed, such as cleaning and keeping the respiratory tract unobstructed, performing endotracheal intubation or tracheotomy, and utilizing a respirator. Meanwhile, oxygen inhalation was given to TBI patients to keep vital signs stable, partial pressure of oxygen (PO_2_), and partial pressure of carbon dioxide (PCO_2_) levels within a normal range.

#### Normal Group

Inclusion criteria were normal people without a history of TBI and aged 15–65. Exclusion criteria were: (1) suffering from nervous system diseases (e.g., cerebrovascular diseases, intracranial space-occupying lesions, encephalitis, psychosis, or dementia, etc.); (2) having respiratory and circulatory system diseases that severely affect cerebral oxygen saturation; (3) with use of drugs that may affect cerebral oxygen saturation.

### APOE Genotype Identification

**Figure 1:**
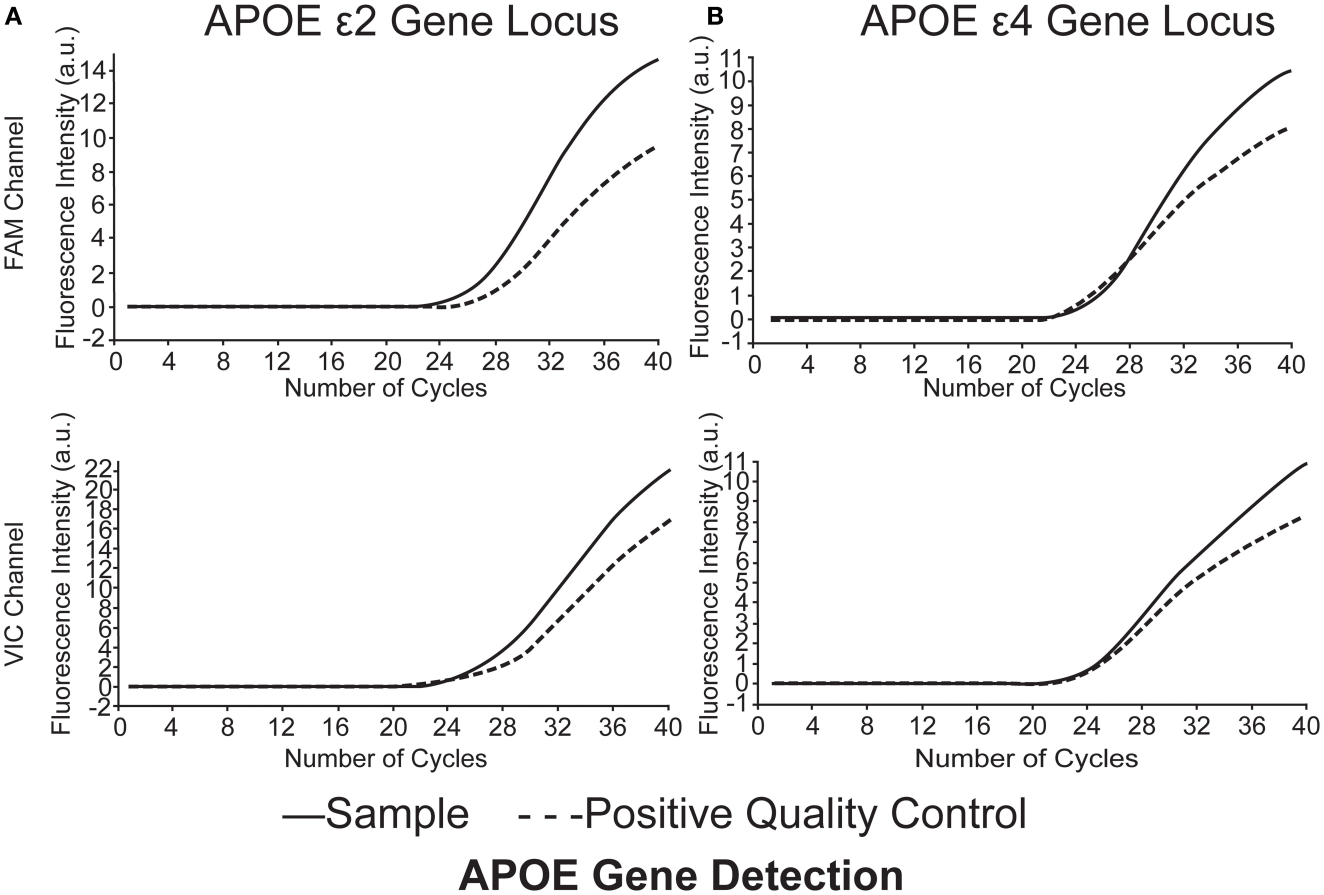
*APOE* genotype of the subjects was identified as ε*2/*ε*4*. **(A)** *APOE*ε*2* gene locus FAM channel (+) VIC channel (+), **(B)** *APOE*ε*4* gene locus FAM channel (+) VIC channel (+). Polymerase chain reaction multiple fluorescence quantitative determination (QF-PCR) was utilized in the present study.

Quantitative fluorescent polymerase chain reaction (QF-PCR) was used to determine the *APOE* genotype of the subjects. The steps were as follows: (1) 2 ml venous blood was collected from the subject to extract the DNA by using the QIAGEN DNA extraction kit; (2) 2 ul genomic DNA of the sample was added into the reaction tube containing 4 kinds of PCR reaction solutions by using the human *APOE* gene detection kit (Wuhan Youzhiyou Medical Technology Co., Ltd.); (3) the PCR reaction tube was removed to the nucleic acid amplification area for detection; and (4) determine the *APOE* genotype (Table 1 and Figure 1).

**Table 1:** APOE genotype determination.

**Signal channel**		**Genotype**	**Gene Loci**
***APOEε2***	***APOEε4***		
VIC	FAM	*ε2/ε2*	526T/T,388T/T
FAM/VIC	FAM	*ε2/ε3*	526C/T,388T/T
FAM/VIC	FAM/VIC	*ε2/ε4*	526C/T,388T/C
FAM	FAM	*ε3/ε3*	526C/C,388T/T
FAM	FAM/VIC	*ε3/ε4*	526C/C,388T/C
FAM	VIC	*ε4/ε4*	526C/C,388C/C

### Monitoring of rScO_2_

MINR-P100 non-invasive cerebral blood oxygen monitor (Chongqing Mingxi Medical Equipment Co., Ltd.) was used in the present study to monitor rScO_2_. For TBI patients, the monitoring of rScO_2_ by NIRS was performed during 1–3 days after TBI. Meanwhile, NIRS was also used immediately to monitor the rScO_2_ of patients once their condition changed. For normal people, the monitoring of rScO_2_ by NIRS was performed under a relaxed and awake condition. The forehead skin was exposed and cleaned, and the probes were closely attached and fixed to bilateral forehead skin. The probes were located 1–2 cm from the upper edge of the eyebrow arch, then rScO_2_ data was detected and collected. The average monitoring time was 30 min (Standard division, ±0.5) for each patient, then an average rScO_2_ value was obtained from the NIRS machine.

### Statistical Analysis

The SPSS 25.0 statistical software was applied in the study. Between-group differences in rScO_2_ value were tested with the use of Student's *t*-tests, and differences in categorical outcomes with the use of Chi-square (χ^2^) tests. Single-factor and multifactor logistic regression analysis were applied to analyze independent risk factors affecting the change of rScO_2_, and *P* < 0.05 was considered statistically significant.

## Result

A total of 176 consecutive TBI patients were collected in this study, and 8 patients were excluded according to the inclusion and exclusion criteria. Therefore, 168 subjects including 114 TBI patients and 54 normal people were enrolled.

### Genotype Distribution

*APOE* has three alleles (*APOE*ε*2, APOE*ε*3, and APOE*ε*4*), which encode six phenotypes including three homozygotes (ε*2/2*, ε*3/*ε*3*, ε*4/*ε*4*) and three heterozygotes (ε*2/*ε*3*, ε*3/*ε*4*, ε*2/*ε*4*). The gene distribution and allele frequency of the TBI group and the normal group in this study are consistent with Hardy-Weinberg's law (Table 2).

**Table 2:** *APOE* genotypes and allele frequencies in the TBI group and the normal group.

**Group**	**Genotype (*****n*****)**						**Allele Frequency (%)**		
	***ε2/ε2***	***ε2/ε3***	***ε2/ε4***	***ε3/ε3***	***ε3/ε4***	***ε4/ε4***	***ε2***	***ε3***	***ε4***
TBI Group (*n* = 114)	4	11	4	81	9	5	10.09	79.82	10.09
Normal Group (*n* = 54)	2	4	2	39	5	2	9.26	80.56	10.18

### Clinical Data of Normal Group and TBI Group

A total of 168 patients were included in this study, including 114 TBI patients and 54 normal people. The general clinical data, such as gender, age, smoking, alcohol-drinking, and hypertension, showed no significant difference between ε4 carriers and ε4 non-carriers in both the TBI group and normal group (*P* > 0.05 by Chi-square tests for differences in the composition of these data) (Tables 3, 4).

**Table 3:** Comparison of characteristics in 54 normal subjects and their condition.

**Items**	***APOEε4* non-carriers (*n* = 48)**	***APOEε4* carriers (*n* = 6)**	***P***
Sex			
Male	23	4	0.669
Female	25	2	
Alcohol-drinking			
Yes	20	3	1.000
No	28	3	
Smoking			
Yes	19	1	0.395
No	29	5	

**Table 4:** Comparison of clinical data of ε4 non-carriers and ε4 carriers in TBI patients.

**Items**	***APOE ε4* non-carriers (*n* = 96)**	***ε4* carriers (*n* = 18)**	***P***
Gender			
Male	56	11	0.826
Female	40	7	
Age (year)			
<45	36	4	0.191
45–60	42	9	
>60	18	5	
Alcohol-drinking			
Yes	51	10	0.850
No	45	8	
Smoking			
Yes	28	9	0.083
No	68	9	
Hypertension			
Yes	29	7	0.467
No	67	11	
Diabetes			
Yes	16	4	0.570
No	80	14	
Hemoglobin (g/L)			
<120	18	2	0.848
120–150	53	12	
>150	25	4	

### rScO_2_ in TBI Group and Normal Group

#### Comparison of rScO_2_ Between TBI Group and Normal Group

**Figure 2:**
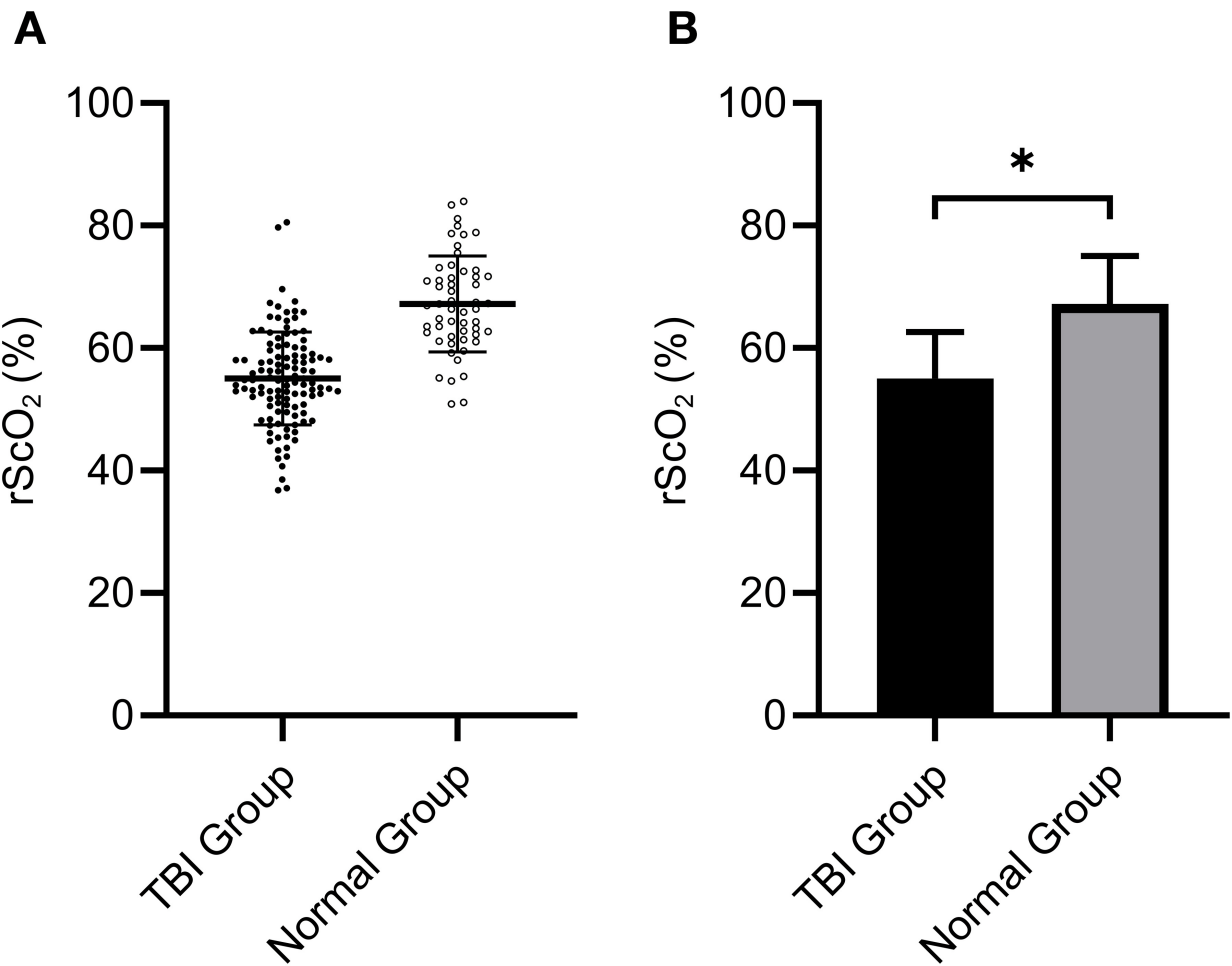
The rScO_2_ (55.06 ± 7.60)% of the TBI group was significantly lower than that of the normal group (67.21 ± 7.80)% (*P < 0.05). **(A)** Shows the distribution of data in two groups, **(B)** indicates the mean ± standard division.

The rScO_2_ data were compared between the TBI group and the normal group by *t*-test. We found that the mean rScO_2_ of TBI patients was (55.06 ± 7.60)%, which was significantly lower than that ((67.21 ± 7.80)%) of the normal group (*P* < 0.001 by Student's *t*-test, Figure 2). This indicated that the rScO_2_ of patients in the early stage of TBI was significantly lower than that of normal people.

#### Independent Risk Factors Affecting rScO_2_ in the TBI Group

In the present study, patients with rScO_2_ below 55% (29 patients) were considered having hypoxia (19), while patients with rScO_2_ above 55% were considered non-hypoxia (85 patients). Single-factor logistic regression analysis showed *APOE*ε*4* (*P* < 0.001), GCS (*P* = 0.001), Marshall CT Class (*P* = 0.013), and hypertension (*P* = 0.002) were independent risk factors for decreased rScO_2_ after TBI. Furthermore, multifactor logistic regression analysis also showed that *APOE*ε*4* (*P* = 0.013, *OR* = 6.742, 95% *CI* = 1.364–30.125), GCS (*P* = 0.041, *OR* = 4.591, 95% *CI* = 1.587–19.512), Marshall CT Class (*P* = 0.007, *OR* = 7.140, 95% *CI* = 0.775–22.145) and hypertension (*P* = 0.023, *OR* = 4.462, 95% *CI* = 1.228–16.239) were independent risk factors that related to the decrease of rScO_2_ after TBI (Table 5).

**Table 5:** Independent risk factors affecting rScO_2_ in the TBI group by single-factor and multi-factor logistic regression analysis.

**Items**	**Non-Hypoxia**	**Hypoxia**	**Single-factor logistic regression (*P*)**	**Multi-factor logistic regression analysis**	
	**(*n* = 85)**	**(*n* = 29)**		***P***	***OR* (95% *CI*)**
Gender			0.184		
Male	53	14			
Female	32	15			
Age (year)			0.936		
<45	31	10			
45–60	36	14			
>60	18	5			
Genotype			<0.001	0.013	6.742 (1.364–30.125)
*APOEε4* carriers	7	11			
*APOEε4* non-carriers	78	18			
Injury mechanism			0.639		
Striking injury	22	7			
Traffic injury	32	14			
Falling or others	31	8			
GCS			0.001	0.041	4.591 (1.587–19.512)
<8	16	10			
9–12	26	15			
13–15	43	4			
Marshall CT Class			0.013	0.007	7.140 (0.775–22.145)
I (Normal CT)	12	1			
II (cisterns present, shift <5 mm)	20	2			
III (cisterns compressed, shift <5 mm)	11	2			
IV (shift>5 mm)	9	10			
V (evacuated mass)	21	6			
VI (non-evacuated mass)	12	8			
Smoking			0.075		
Yes	23	13			
No	62	16			
Alcohol-drinking			0.835		
Yes	45	15			
No	40	14			
Hypertension			0.002	0.023	4.462 (1.228–16.239)
Yes	20	16			
No	65	13			
Diabetes			0.280		
Yes	13	7			
No	72	22			
Hemoglobin (g/L)			0.371		
<120	16	4			
120–150	49	16			
>150	20	9			

#### Effects of APOE Gene Polymorphism on Early rScO_2_ in TBI Patients

**Figure 3:**
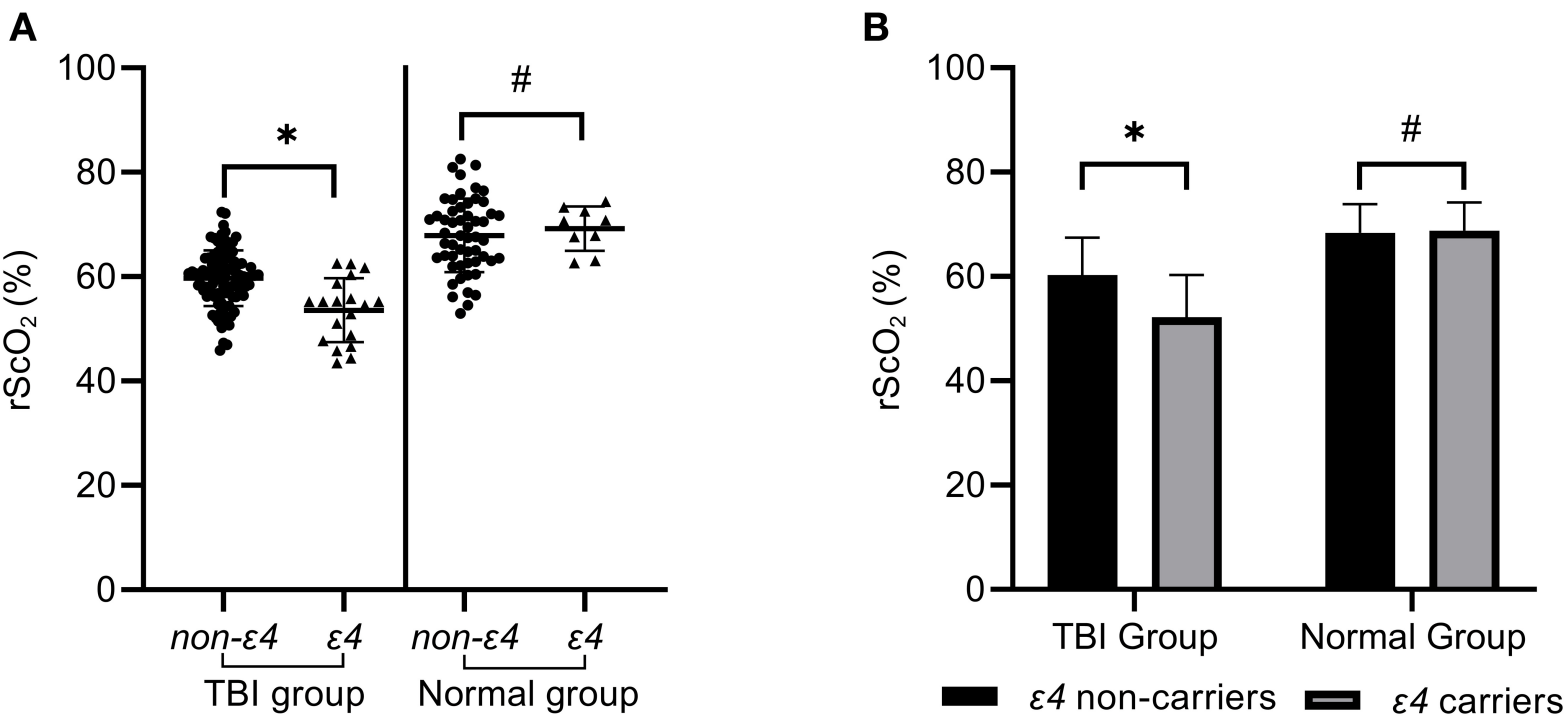
The rScO_2_ of ε*4* carriers in the TBI group was significantly lower than that of ε*4* non-carriers, which was statistically significant (*P < 0.05). However, the rScO_2_ of ε*4* carriers in the normal control group did not show a significant decrease compared with ε*4* non-carriers (^#^*P* > 0.05) in **(A,B)**.

According to Table 5, *APOE*ε*4* was an independent risk factor that affected rScO_2_ of patients in the early stage of TBI. To further study the influence of *APOE*ε*4* on rScO_2_, both TBI patients and normal people were divided into ε*4* non-carriers group and ε*4* carriers group. Statistical analysis showed that, in the early stage of TBI, the rScO_2_ [(52.23±8.02)%] of ε*4* carriers was remarkably lower than that of ε*4* non-carriers [(60.33 ± 7.12)%], which was statistically significant (*P* < 0.001 by Student's *t*-test). Meanwhile, the rScO_2_ of ε*4* non-carriers and ε*4* carriers in the normal group was (68.37 ± 5.56) and (68.75 ± 5.49)% (Table 6 and Figure 3) respectively, indicating no significant difference (*P* > 0.05 by Student's *t*-test).

**Table 6:** Mean rScO_2_ of ε*4* Non-carriers and ε4 Carriers in TBI group and normal group.

**Group**	**rScO**_****2****_ (%)^*^		***P*_**1**_**	**Hypoxia**		***P_**2**_***
	***ε4* non-carriers**	***ε4* carriers**		***ε4* non-carriers**	***ε4* carriers**	
TBI	60.33 ± 7.12	52.23 ± 8.02	<0.001	18/95 (18.95%)	11/19 (57.89%)	0.001
Normal	68.37 ± 5.56	68.75 ± 5.49	0.851	2/45 (4.44%)	1/9 (11.11%)	0.428

* *Mean ± standard division*.

Furthermore, in the TBI group, up to 57.89% (11 of 19) ε4 carriers had hypoxia, which was significantly higher than that (18.95%, 18 of 95) of ε*4* non-carriers (*P* = 0.001 by Chi-square tests). However, in the normal group, only 11.11% (1of 9) ε*4* carriers and 4.44% (2 of 45) ε*4* non-carriers showed hypoxia, which showed no statistical significance (*P* = 0.428 by Chi-square tests) (Table 6).

## Discussion

### TBI and Cerebral Oxygen Metabolism

As shown in the results, the mean rScO_2_ of TBI patients (55.06 ± 7.60)% in the early stage of TBI was significantly lower than that of normal people (67.21 ± 7.80)% (*P* < 0.05), which suggested that the rScO_2_ of patients significantly decreased in the early stage of TBI. Cerebral blood flow often decreased after TBI, which may lead to ischemia and hypoxia in brain tissue and result in irreversible brain damage. Relevant literature reported that more than 90% of people who died of TBI may have secondary cerebral ischemia and hypoxia (20). Similarly, the results of this study also showed the rScO_2_ of patients was significantly decreased in the early stage of TBI as compared with normal people. Furthermore, the rScO_2_ was considered abnormal when it was below 55%, and an over 12% decrease in rScO_2_ usually indicated the possibility of cerebral ischemia, which needs clinical intervention (7, 19, 21). Therefore, timely and accurate monitoring of rScO_2_ is extremely important for TBI patients in the NICU.

Presently, multiple methods including NIRS are used to monitor and evaluate the condition of TBI patients in the NICU, such as brain tissue oxygen tension (PbtO2), jugular venous oxygen saturation (SvjO_2_), cerebral microdialysis, thermal diffusion measurement of cerebral blood flow, and electroencephalogram (EEG) (22). As compared with PbtO_2_ and SvjO_2_, NIRS is non-invasive, continuous, more convenient and safe. In the NICU, the cerebral oxygen metabolism can be monitored by NIRS continuously, which shows not only the dynamic changes of rScO_2_ in real-time but also the mean rScO_2_ (23, 24). In our NICU, NIRS is a routine method to monitor the rScO_2_ of patients, which provides important information about cerebral oxygen metabolism to us in real-time. Proposed by Franz Jobsis in 1977, NIRS is based on the permeability of biological tissues to near-infrared spectrum (wavelength 700–1,000 nm) and different absorbed light waves for chromophores, such as hemoglobin and reduced hemoglobin, to achieve continuous and non-invasive monitoring of human tissue oxygenation saturation. It has been found that the optical properties of brain tissue will change when cerebral hemorrhage and cerebral ischemia occur. Zweifel et al. observed that the increase of rScO_2_ is consistent with the relief of vasospasm and the improvement of clinical symptoms through arterial imaging, and the arterial spasm is significantly related to the decrease of rScO_2_ on the same side, which suggested that changes of rScO_2_ may reflect the severity of TBI (25). In this study, we also found the level of GCS and Marshall CT Class were the risk factors affecting rScO_2_ in the early stage of TBI, indicating that rScO_2_ was related to the severity of TBI.

### APOE Gene Polymorphism and Cerebral Oxygen Saturation

Previously, we have first shown that, in the cohort of mainland Chinese patients, *APOE*ε*4* carriers were more prone to clinical deterioration in the acute phase after TBI as compared with *APOE*ε*4* non-carriers (16). To further explore the influence of *APOE* on TBI outcomes, a series of studies were carried out in both the clinic and laboratory. In the present clinical study, we used NIRS to monitor rScO_2_ of TBI patients in the NICU. Through single-factor and multifactor logistic analysis, we found *APOE*ε*4* is an independent risk factor that caused the early decline of rScO_2_ in TBI patients. To further verify this speculation, the rScO_2_ of *APOE*ε*4* carriers and non-carriers both in the TBI group and the normal group were analyzed. The results showed that the mean rScO_2_ of *APOE*ε*4* carriers in TBI patients was significantly lower than that of *APOE*ε*4* non-carriers in the early stage of TBI. Furthermore, in TBI patients, decreased rScO_2_ was found in 57.89% *APOE*ε*4* carriers, but only in 18.95% *APOE*ε*4* non-carriers, indicating the rScO_2_ of patients with *APOE*ε*4* were more likely to decrease as compared to patients without *APOE*ε*4* in the early stage of TBI.

Meanwhile, by setting normal people as control, we found that there was no significant difference of rScO_2_ between *APOE*ε*4* carriers and non-carriers in the normal group [(68.75 ± 5.49)% vs. (68.37 ± 5.56)%, *P* = 0.851], indicating *APOE*ε*4* leads to a decrease of rScO_2_ in TBI patient but not in normal people. In another word, the negative effect of *APOE*ε*4* on rScO_2_ can be induced by TBI, which was similar to our previous results.

By monitoring the EEG of TBI patients in the NICU, we have found that *APOE*ε*4* is a risk factor for the worsening EEG activity at the acute phase (26). Additionally, we also found *APOE*ε*4* induced cerebral hypoperfusion which may directly cause impairment of cerebral oxygen metabolism in the early phase of aSAH (26). Furthermore, in the metabolic/hemodynamic model (MHM) coupling neuronal activity with EEG and hemodynamic responses, Sotero et al. (27) also confirmed that inhibitory or excitatory activity was accompanied by reductions or increase of oxygen consumption, cerebral blood flow (CBF), and blood oxygenation level-dependent (BOLD) responses, indicating EEG is very sensitive to cerebral ischemia and hypoxia. Studies on cerebral oxygen metabolism have also suggested the EEG had a positive correlation with cerebral oxygen saturation (28, 29), which was consistent with our studies.

In the laboratory researches, we found *APOE*ε*4* may affect intracellular calcium concentration, inflammatory response, excitatory amino acid release, and neuron apoptosis after mechanical injury (30, 31). Furthermore, we also found that by modulating NF-κB/MMP-9 pathway (17), *APOE*ε*4* may affect blood-brain barrier permeability which plays an important role in the brain edema and cerebral oxygen metabolism. Therefore, we speculate that *APOE*ε*4* may affect the cerebral oxygen saturation of TBI patients through the above process, and then result in worse EEG and clinical outcome eventually. Besides, some negative effects of *APOE*ε*4* will not be reflected under normal physiological conditions but will be induced under pathological conditions such as TBI, affecting the prognosis of TBI patients. However, more studies on the mechanism through which *APOE*ε*4* influences the rScO_2_ and outcome of TBI patients are still needed in the future.

We have to acknowledge, as a method monitoring the condition of cerebral oxygen saturation, NIRS has several limitations including, (1) the result of NIRS reflects the corresponding changes in the monitoring process, so the absolute value of rScO_2_ can't be obtained through NIRS; (2) the result of rScO_2_ monitored by NIRS may be influenced by factors including blood pressure, blood oxygen saturation, some narcotic drugs, etc.; (3) the interpretation of NIRS result may be influenced by the experience of the operators. Besides, this is a small sample and single-center clinical study, which has its limitations and requires large-scale and multiple centers for further verification. We will continue to carry out additional researches to explore the possible mechanism.

## Conclusion

NIRS is a non-invasive and convenient method to evaluate rScO_2_ of TBI patients in the NICU. The rScO_2_ may be significantly decreased after TBI. Furthermore, *APOE*ε*4* may be a risk factor for decreased rScO_2_ in the early stage of TBI, which may be a possible basis to develop more precise and individualized treatments for different patients.

## Data Availability Statement

The data analyzed in this study is subject to the following licenses/restrictions: The datasets analyzed in this article are not publicly available. Requests to access these datasets should be directed to drjiangli2019@163.com.

## Ethics Statement

The studies involving human participants were reviewed and approved by Ethics Committee of the First Affiliated Hospital of Chongqing Medical University. The patients/participants provided their written informed consent to participate in this study.

## Author Contributions

This study was designed and managed by YX and LJ, with data collected and processed by SX and ZW. Data were analyzed by WD and YZ. The manuscript was prepared by SX, ZW, YX, XS, QS, and LJ. All authors contributed to the article and approved the submitted version.

## Conflict of Interest

The authors declare that the research was conducted in the absence of any commercial or financial relationships that could be construed as a potential conflict of interest. The reviewer YW declared a shared affiliation with the authors to the handling editor at time of review.

## References

[1] Kowalski RG Haarbauer-Krupa JK Bell JM Corrigan JD Hammond FM Torbey MT Acute ischemic stroke after moderate to severe traumatic brain injury. Stroke. (2017) 48:1802–9. 10.1161/STROKEAHA.117.017327 28611087 PMC6025795

[2] Shao X Hu Q Chen S Wang Q Xu P Jiang X. Ghrelin ameliorates traumatic brain injury by down-regulating bFGF and FGF-BP. Front Neurosci. (2018) 12:445. 10.3389/fnins.2018.00445 30026681 PMC6041414

[3] Mohamadpour M Whitney K Bergold PJ. The importance of therapeutic time window in the treatment of traumatic brain injury. Front Neurosci. (2019) 13:07. 10.3389/fnins.2019.00007 30728762 PMC6351484

[4] Veenith TV Carter EL Geeraerts T Grossac J Newcombe VFJ Outtrim J. Pathophysiologic mechanisms of cerebral ischemia and diffusion hypoxia in traumatic brain injury. JAMA Neurol. (2016) 73:542–50. 10.1001/jamaneurol.2016.0091 27019039

[5] Zhang L Zhang L Liu H Jiang F Wang H Li D. Inhibition of Epac2 attenuates neural cell apoptosis and improves neurological deficits in a rat model of traumatic brain injury. Front Neurosci. (2018) 12:263. 10.3389/fnins.2018.00263 29740274 PMC5924794

[6] Robertson CS Valadka AB Hannay HJ Contant CF Gopinath SP Cormio M. Prevention of secondary ischemic insults after severe head injury. Crit Care Med. (1999) 27:2086–95. 10.1097/00003246-199910000-00002 10548187

[7] Maas AIR Stocchetti N Bullock R. Moderate and severe traumatic brain injury in adults. Lancet Neurol. (2008) 7:728–41. 10.1016/S1474-4422(08)70164-9 18635021

[8] Rosenthal G Furmanov A Itshayek E Shoshan Y Singh V. Assessment of a noninvasive cerebral oxygenation monitor in patients with severe traumatic brain injury. J Neurosurg. (2014) 120:901–7. 10.3171/2013.12.JNS131089 24484228

[9] Cooper RJ Selb J Gagnon L Phillip D Schytz HW Iversen HK. A systematic comparison of motion artifact correction techniques for functional near-infrared spectroscopy. Front Neurosci. (2012) 6:147. 10.3389/fnins.2012.00147 23087603 PMC3468891

[10] Guhathakurta D Dutta A. Computational pipeline for NIRS-EEG joint imaging of tDCS-evoked cerebral responses—an application in ischemic stroke. Front Neurosci. (2016) 10:261. 10.3389/fnins.2016.00261 27378836 PMC4913108

[11] Naguib AN Winch PD Sebastian R Gomez D Guzman L Rice J. The correlation of two cerebral saturation monitors with jugular bulb oxygen saturation in children undergoing cardiopulmonary bypass for congenital heart surgery. J Intensive Care Med. (2017) 32:603–8. 10.1177/0885066616663649 27530512

[12] Goldman S Sutter F Ferdinand F Trace C. Optimizing intraoperative cerebral oxygen delivery using noninvasive cerebral oximetry decreases the incidence of stroke for cardiac surgical patients. Heart Surgery Forum. (2004) 7:E376–81. 10.1532/HSF98.20041062 15799908

[13] Liaquat I Dunn LT Nicoll JA Teasdale GM Norrie JD. Effect of apolipoprotein E genotype on hematoma volume after trauma. J Neurosurg. (2002) 96:90–6. 10.3171/jns.2002.96.1.0090 11795256

[14] McCarron MO Weir CJ Muir KW Hoffmann KL Graffagnino C Nicoll JA. Effect of apolipoprotein E genotype on in-hospital mortality following intracerebral haemorrhage. Acta Neurol Scand. (2003) 107:106–9. 10.1034/j.1600-0404.2003.01365.x 12580859

[15] Millar K Nicoll JA Thornhill S Murray GD Teasdale GM. Long term neuropsychological outcome after head injury: relation to APOE genotype. J Neurol Neurosurg Psychiatr. (2003) 74:1047–52. 10.1136/jnnp.74.8.1047 12876232 PMC1738588

[16] Jiang Y Sun X Xia Y Tang W Cao Y Gu Y. Effect of APOE polymorphisms on early responses to traumatic brain injury. Neurosci Lett. (2006) 408:155–8. 10.1016/j.neulet.2006.08.082 16997460

[17] Teng Z Guo Z Zhong J Cheng C Huang Z Wu Y. ApoE Influences the blood-brain barrier through the NF-kappaB/MMP-9 pathway after traumatic brain injury. Sci Rep. (2017) 7:6649. 10.1038/s41598-017-06932-3 28751738 PMC5532277

[18] Levin HS Diaz-Arrastia RR. Diagnosis, prognosis, and clinical management of mild traumatic brain injury. Lancet Neurol. (2015) 14:506–17. 10.1016/S1474-4422(15)00002-2 25801547

[19] McCormick PW Stewart M Ray P Lewis G Dujovny M Ausman JI. Measurement of regional cerebrovascular haemoglobin oxygen saturation in cats using optical spectroscopy. Neurol Res. (1991) 13:65–70. 10.1080/01616412.1991.11739967 1675450

[20] Ko SB. Multimodality monitoring in the neurointensive care unit: a special perspective for patients with stroke. J Stroke. (2013) 15:99–108. 10.5853/jos.2013.15.2.99 24324945 PMC3779668

[21] Schewe JC Thudium MO Kappler J Steinhagen F Eichhorn L Erdfelder F. Monitoring of cerebral oxygen saturation during resuscitation in out-of-hospital cardiac arrest: a feasibility study in a physician staffed emergency medical system. Scand J Trauma Resusc Emerg Med. (2014) 22:58. 10.1186/s13049-014-0058-y 25286829 PMC4196010

[22] Grinspan ZM Pon S Greenfield JP Malhotra S Kosofsky BE. Multimodal monitoring in the pediatric intensive care unit: new modalities and informatics challenges. Semin Pediatr Neurol. (2014) 21:291–8. 10.1016/j.spen.2014.10.005 25727511

[23] Brawanski A Faltermeier R Rothoerl RD Woertgen C. Comparison of near-infrared spectroscopy and tissue p(O2) time series in patients after severe head injury and aneurysmal subarachnoid hemorrhage. J Cereb Blood Flow Metab. (2002) 22:605–11. 10.1097/00004647-200205000-00012 11973433

[24] Bhatia R Hampton T Malde S Kandala NB Muammar M Deasy N. The application of near-infrared oximetry to cerebral monitoring during aneurysm embolization: a comparison with intraprocedural angiography. J Neurosurg Anesthesiol. (2007) 19:97–104. 10.1097/ANA.0b013e318031376d 17413995

[25] Zweifel C Castellani G Czosnyka M Helmy A Manktelow A Carrera E. Noninvasive monitoring of cerebrovascular reactivity with near infrared spectroscopy in head-injured patients. J Neurotr. (2010) 27:1951–8. 10.1089/neu.2010.1388 20812789

[26] Jiang L Yin X Yin C Zhou S Dan W Sun X. Different quantitative EEG alterations induced by TBI among patients with different APOE genotypes. Neurosci Lett. (2011) 505:160–4. 10.1016/j.neulet.2011.10.011 22015765

[27] Sotero RC Trujillo-Barreto NJ. Biophysical model for integrating neuronal activity, EEG, fMRI and metabolism. Neuroimage. (2008) 39:290–309. 10.1016/j.neuroimage.2007.08.001 17919931

[28] Hoshi Y Kosaka S Xie Y Kohri S Tamura M. Relationship between fluctuations in the cerebral hemoglobin oxygenation state and neuronal activity under resting conditions in man. Neurosci Lett. (1998) 245:147–50. 10.1016/S0304-3940(98)00197-9 9605477

[29] Roche-Labarbe N Wallois F Ponchel E Kongolo G Grebe R. Coupled oxygenation oscillation measured by NIRS and intermittent cerebral activation on EEG in premature infants. Neuroimage. (2007) 36:718–27. 10.1016/j.neuroimage.2007.04.002 17482837

[30] Jiang L Zhong J Dou X Cheng C Huang Z Sun X. Effects of ApoE on intracellular calcium levels and apoptosis of neurons after mechanical injury. Neuroscience. (2015) 301:375–83. 10.1016/j.neuroscience.2015.06.005 26073697

[31] Jiang Y Sun X Gui L Xia Y Tang W Cao Y. Correlation between APOE−491AA promoter in epsilon4 carriers and clinical deterioration in early stage of traumatic brain injury. J Neurotra. (2007) 24:1802–10. 10.1089/neu.2007.0299 18159991

